# The Influence of Endothelial Function and Myocardial Ischemia on Peak Oxygen Consumption in Patients with Coronary Artery Disease

**DOI:** 10.1155/2012/274381

**Published:** 2012-10-11

**Authors:** Simon L. Bacon, Andrew Sherwood, Alan Hinderliter, Annik Plourde, Lee Pierson, James A. Blumenthal

**Affiliations:** ^1^Montreal Behavioural Medicine Centre, Hôpital du Sacré-Coeur de Montréal, A University of Montreal Affiliated Hospital, Montréal, QC, Canada H4J 1C5; ^2^Department of Exercise Science, Concordia University, Montreal, QC, Canada H4B 1R6; ^3^Research Centre, Hôpital du Sacré-Coeur de Montréal, A University of Montreal Affiliated Hospital, Montréal, QC, Canada H4J 1C5; ^4^Research Centre, Montreal Heart Institute, A University of Montreal Affiliated Hospital, Montréal, QC, Canada H1T 1C8; ^5^Department of Psychiatry and Behavioral Science, Duke University Medical Center, Durham, NC 27708, USA; ^6^Department of Medicine, University of North Carolina at Chapel Hill, Chapel Hill, NC 27599, USA; ^7^Department of Psychology, Université du Québec à Montréal, Montréal, QC, Canada H3C 3P8

## Abstract

Impaired endothelial function has been shown to limit exercise in coronary artery disease (CAD) patients and has been implicated in myocardial ischemia. However, the association of endothelial function and ischemia on peak exercise oxygen consumption (VO_2_) has not been previously reported. A total of 116 CAD patients underwent standard exercise stress testing, during which VO_2_ was measured. On a separate day, endothelial-dependent and -independent function were assessed by ultrasound using flow-mediated arterial vasodilation (FMD) and sublingual glyceryl trinitrate administration (GTNMD) of the brachial artery. Patients with exercise-induced myocardial ischemia had lower FMD than nonischemic patients (3.64 ± 0.57 versus 4.98 ± 0.36, *P* = .050), but there was no difference in GTNMD (14.11 ± 0.99 versus 15.47 ± 0.63, *P* = .249). Analyses revealed that both FMD (*P* = .006) and GTNMD (*P* = .019) were related to peak VO_2_. However, neither the presence of ischemia (*P* = .860) nor the interaction of ischemia with FMD (*P* = .382) and GTNMD (*P* = .151) was related to peak VO_2_. These data suggest that poor endothelial function, potentially via impaired NO production and smooth muscle dysfunction, may be an important determinant of exercise capacity in patients with CAD, independent of myocardial ischemia.

## 1. Introduction

Under normal physiological conditions, exercise-induced increases in myocardial oxygen demand are met by coronary vasodilation and increased oxygen delivery, thus preventing ischemia or perfusion defects [1]. Vasodilation during exercise is, in part, contingent upon the vascular endothelium [2, 3] and endothelial dysfunction may limit skeletal muscle oxygen delivery and exercise capacity. For example, impaired endothelial function has been associated with poorer exercise treadmill time in CAD patients [4] and in postcardiac transplant patients [5]. 

Endothelial dysfunction is widely implicated in cardiovascular disease and its complications [6]. Compared to matched controls, patients with coronary artery disease (CAD) have a greater degree of endothelial dysfunction [7, 8]. In addition, patients with exercise-induced myocardial ischemia have impaired endothelial function compared to nonischemic patients [4]. It is possible that endothelial dysfunction may result in failure of arterial vasodilation during exercise [9], thus compromising oxygen supply and precipitating ischemia in a CAD population [10].

The primary aim of the current study was to assess impact of exercise-induced myocardial ischemia on the relationship between endothelial function, as measured by flow mediated dilatation in the brachial artery (FMD) and peak oxygen consumption (VO_2_) during symptom limited exercise testing in stable CAD patients. It was hypothesized that endothelial function would be positively correlated to VO_2_, that patients who experienced ischemia during exercise would have greater degrees of endothelial dysfunction, and that there would be a significant interaction between ischemia and endothelial function in the prediction of VO_2_, such that the relationship between endothelial function and VO_2_ would be strongest in patients without ischemia.

## 2. Materials and Methods

### 2.1. Participants

Patients were participants in a randomized controlled trial, the results of which have been previously reported [11–13]. Patients were included in the current secondary analyses if they had complete endothelial function and exercise testing data at baseline. As such, of the 145 patients recruited, a total of 116 patients were used in the current analyses. Patients not included in the analyses did not differ from those included on age, sex, medication status, or medical comorbidities. All patients that were recruited had documented coronary artery disease with prior evidence of inducible exercise ischemia. Patients were excluded from the study if they had baseline blood pressure >200/120 mm Hg, LVEF <30%, history of MI, CABG, or PCI in the last 3 months, or had severe electrophysiological disorders. The study was approved by the Institutional Review Boards of both Duke University Medical Center and the Durham Veterans Affairs Medical Center. Signed informed consent was obtained from all patients prior to their participation. None of the authors have any conflict of interests or competing interests with the content of the paper.

### 2.2. Measurement of Endothelial Function

Participants were removed from all antianginal medications at least 48 hrs prior to testing, with the exception of 23 patients who remained on medications (21 beta-blockers, 9 long-acting nitrates, and 7 calcium channel blockers) due to adverse cardiac profiles. To control for this potential confounder, being on or off medications during the endothelial function test was included in the analyses as a covariate. During the morning following at least an 8 hour fast, endothelial function was assessed in participants using flow-mediated arterial vasodilation (for further details see [14]). The technique, which conforms to published guidelines [15], involves the digital capture and storage of high-resolution end-diastolic (ECG R-wave synchronized) longitudinal B-mode images of the brachial artery. Images were obtained at rest, during reactive hyperemia induced by 5 minutes of forearm ischemia, and following sublingual glyceryl trinitrate administration (GTNMD) using an ultrasound imaging system with a 7–11 MHz linear-array transducer and an Aspen acquisition system (Acuson, Mountain View, California). 

### 2.3. Graded Exercise Test

On a separate visit and after resumption of all antianginal medications (for which 16 participants were not taking antianginal medications), participants performed a maximal graded exercise test on a motor-driven treadmill (Quinton, model Q5000) using the Duke-Wake Forrest protocol [16]. The electrocardiogram was continuously monitored at rest, during exercise, and throughout recovery with a 12-lead trace being printed at 1-minute intervals. Blood pressure was measured at rest and during each workload with an automated BP monitor (Accutracker model 4240; Suntech). Oxygen consumption (VO_2_) was measured during exercise by respiratory gas exchange analysis with a calibrated metabolic cart (Sensormedics, model 2900; Yorba Linda, CA), using a breath-by-breath technique. VO_2_ data were averaged over 20 second intervals, and peak VO_2_ was taken as the highest 20 second average during the last three minutes of the test. Every minute during exercise patients were asked to rate chest pain (1–10 scale) and perceived exertion (Borg 6–20 scale), by pointing to hand held charts. Ischemia was determined by a senior cardiologist and was defined as ST depression >1.0 mm for at least 3 beats. All electrocardiogram readings were made by an experienced senior cardiologist.

### 2.4. Data Analysis

Student *t*-tests, *χ* ^2^ analyses, and general linear models (used when covariates were required) were used to assess differences in demographic and exercise variables between patients who had exercise-inducible ischemia and those who did not exhibit ischemia during the exercise test. Current hypertensive status was defined by use of antihypertensive medication. All significantly different demographic and exercise variables were used as covariates in the multivariate analyses. As BMI was significantly different between the groups and was entered into the main models, absolute peak VO_2_ (L/min) and not peak VO_2_ relative to body weight (mL/kg/min) were used for statistical purposes. This was done to prevent body weight being adjusted for twice. Conventional general linear models were used to test the relationships between endothelial function, exercise performance, and ischemia. In total four models were estimated: model 1 tested the independent effects of FMD and ischemia on peak VO_2_ during the exercise test; model 2 tested the joint effect of FMD and ischemia on peak VO_2_; model 3 tested the independent effects of GTNMD and ischemia on peak VO_2_ during the exercise test; model 4 tested the joint effect of GTNMD and ischemia on peak VO_2_. All models used brachial artery vessel size, age, medication status during the FMD test, and BMI as covariates. All statistical analyses were performed using SAS (version 8.2, Cary, NC), with significance set at *P* < .05.

## 3. Results and Discussion

### 3.1. Clinical Characteristics

The clinical characteristics of the 34 patients who exhibited exercise inducible ischemia and the 84 patients who were not ischemic are summarized in Table 1. The ischemic group was significantly older and had a significantly lower BMI compared to the nonischemic group. There were no differences in any other characteristics, including resting blood pressure and heart rate, as measured in a seated position prior to the start of the stress test. FMD, controlling for vessel size, was significantly lower in the ischemic group, whereas there was no significant difference in GTNMD between the groups.  Table 2 shows that there were no significant differences in exercise treadmill time, chest pain, or level of perceived exertion between the two groups.

### 3.2. Relationship between Endothelial Function, Ischemia, and Peak VO_2_

**Figure 1:**
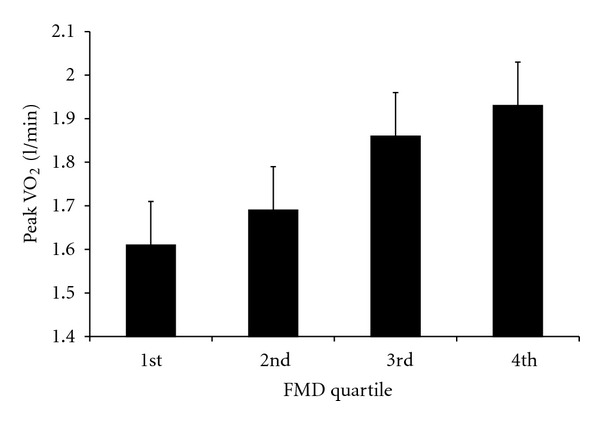
The relationship between endothelial function (quartiles of flow-mediated dilatation: FMD) and peak oxygen uptake (Peak VO_2_), adjusting for vessel size, age, and body mass index.

The results of the sequence of general linear models for peak VO_2_ are presented in Table 3, with the parameter estimate b (analogous to unstandardized regression coefficients) and *P*-values shown for each model. The results for Models 1 and 2 revealed that of the covariates, age and brachial artery vessel size were related to peak VO_2_ during exercise. From Model 1, we observed a main effect for FMD, but not ischemia, on peak VO_2_ levels, such that lower levels of FMD were related to lower peak VO_2_ values.  Figure 1 shows the main effect of FMD (split by quartiles) on VO_2_. The FMD × ischemia interaction in Model 2 was not significant, indicating that there was no evidence for a joint effect of these variables on peak VO_2_. A similar pattern of results were seen in Models 3 and 4. Age, BMI, and brachial artery vessel size were all related to peak VO_2_. Model 3 revealed a main effect for GTNMD, and not ischemia, on peak VO_2_, and a nonsignificant interaction between GTNMD and ischemia in Model 4. These results found that reductions in GTNMD were associated with reduced peak VO_2_, and that there were no main or interaction effects of ischemia on peak VO_2_. Of note, these results did not substantially change when those patients who were taking medications during the FMD test were removed from the analyses.

### 3.3. Discussion

The current study found that both endothelial-dependent and -independent vascular reactivity were associated with lower levels of peak VO_2_ in CAD patients undergoing exercise treadmill testing. This relationship remained significant after adjusting for age, BMI, and brachial artery vessel size. However, neither the presence of ischemia during the exercise test nor the interaction of ischemia with FMD or GTNMD was related to peak VO_2_. As such, the major new finding of this study is that endothelial-dependent and -independent vasodilator function is related to exercise capacity independently of whether a patient experiences myocardial ischemia. In line with previous studies [4, 10, 17], we did find that, as a group, ischemic patients had lower FMD values than nonischemic patients. 

Both exercise capacity and endothelial function have been shown to predict clinical events in patients with CAD [18, 19]. Additionally, both have been shown to improve with exercise training [20–24]. Reviews of the previous research in this area has shown that endothelial dysfunction seems to limit exercise capacity and that this may be through multiple mechanisms [2, 3, 25], including altered production of NO [23], prostanoids [26], or endothelin [27]. However, it should be noted that the vast majority of this work has come from healthy participants and animal studies. Our finding that both FMD and GTNMD were related to VO_2_ is consistent with the idea of a multimechanistic process. Previous research has suggested that a significant response or relationship seen with both endothelial-dependent (FMD) and -independent (GTNMD) stimuli, as in the current study, is indicative of both impaired endothelial function and smooth muscle dysregulation [28, 29], and thus, may contribute to the relationship between vasoreactivity and exercise capacity.

It has been suggested that endothelial dysfunction can lead to paradoxical coronary vasoconstriction during exercise, thus helping to precipitate ischemia [30]. Endothelial dysfunction is associated with reduced exercise treadmill time in CAD patients [4] and in postcardiac transplant patients [5]. Therefore, we had anticipated that the relationship between endothelial function and VO_2_ would be weaker or even nonexistent in those patients with ischemia. However, the lack of an interaction between FMD or GTNMD and ischemia indicated that this was not true in the current study.

One inherent limitation of cross-sectional research, as in the current study, is that we are unable to infer directionality of the endothelial function-oxygen consumption relationship. Previous research has suggested that endothelial dysfunction precedes oxygen consumption limitation. For example, an exercise intervention in heart failure patients found that improvements in exercise capacity were proportional to the beneficial changes seen in agonist-mediated endothelial dilation [21]. However, more work is needed to elucidate the true nature of the FMD-VO_2_ relationship. It is possible that some underlying factor, which was not measured in the current study, directly influences both the endothelium and oxygen requirements such that the relationship seen is an artifact of the third variable. For example, elevated C-reactive protein or homocysteinemia have both been shown to influence vasoreactivity [31, 32] and neither were assessed in the current population. Also, we were not able to assess the potential effect of shear rate as this data was not recorded and this may account for some of the observed relationship. 

Patients in the present study were withdrawn from their anti-ischemic medications prior to and during the endothelial function testing but remained on medications during the exercise test. This is likely to account for the relatively low incidence of exercise-induced ischemia seen during the exercise test. It is impossible to truly estimate the residual effects of medications on the outcome measures. However, a previous study found that endothelial function was not altered in patients when they took their normal regimen of cardiac medications compared to when they were medication-free [33]. This study suggests that medication utilization has small effects on endothelial function. Of course, it is also possible that the taking of medications during the exercise stress test may have had a beneficial effect on exercise performance and myocardial perfusion, which may have influenced the results. However, this does reflect what a patient would normally experience during daily living.

Finally, given that men and women seem to have different CAD courses and patterns of CAD outcomes [34, 35] and that endothelial function has been postulated as one reason for such differences [36], it would have been interesting to include further analyses which had a sex interaction or were sex-specific. However, it should be noted that only around 30% of the sample were women, which did not provide enough power for us to conduct such analyses. The need for such future studies in this area is further strengthened by the fact that when we included sex as an additional covariate there was a reduction in the estimates for the effects of FMD (change from 0.04 to 0.02) and GTNMD (change from 0.02 to 0.01). Though it is possible that this reduction in effect seen when sex was added to the model could be a consequence of over-fitting [37], there are other potential physiological explanations, such as the fact that women are thought to have greater microvascular dysfunction relative to men [38, 39]. As the current study did not have the capacity to disentangle such issues further work to assess the sex-specific nature of this relationship should be conducted.

## 4. Conclusions

Endothelial-dependent and -independent vascular dysfunction were associated with reduced oxygen consumption in CAD patients, and these relationships were independent of myocardial ischemia. These data suggest that impaired vascular reactivity via reduced NO production and smooth muscle dysfunction may be an important determinant of exercise capacity in patients with CAD. Further research is needed to delineate the exact mechanisms of these relationships.

## Figures and Tables

**Table 1:** Clinical and demographic variables for those patients with and without exercise-induced ischemia.

M (SD)	Ischemic	Nonischemic	χ^2^/t/F	P
*n*	34	82		
Age (years)	66 (11)	61 (10)	2.26	.026
BMI (kg·m^−2^)	27.9 (4.1)	30.6 (5.3)	2.68	.009
% Women (*n*)	35 (12)	27 (21)	1.11	.293
% On meds during FMD (*n*)	12 (4)	23 (19)	1.97	.161
% Hypertensive (*n*)	41 (14)	60 (49)	3.34	.068
% Dyslipidemic (*n*)	89 (73)	32 (94)	0.73	.394
% Diabetic (*n*)	19 (23)	5 (15)	1.05	.306
% Current smoker (*n*)	12 (15)	3 (9)	0.72	.396
FMD (%)*	3.64 (0.57)	4.98 (0.36)	3.92	.050
GTNMD (%)*	14.11 (0.99)	15.47 (0.63)	1.34	.249

*Corrected M (SE), adjusting for vessel size.

BMI: body mass index, ETT: exercise tolerance test, FMD: flow-mediated dilatation, GTNMD: GTN-mediated dilatation.

**Table 2:** Exercise variables for those patients with and without exercise-induced ischemia.

M (SD)	Ischemic	Non-ischemic	*χ* ^2^/*t*/*F*	*P*
Total ETT time (s)	463 (142)	448 (172)	0.43	.666
Peak VO_2_ (mL·kg^−1^·min^−1^)	19.7 (5.0)	20.1 (6.2)	0.30	.765
Peak VO_2_ abs (L·min^−1^)	1.62 (0.56)	1.83 (0.64)	1.68	.095
Resting SBP (mmHg)	139.5 (18.7)	136.7 (19.4)	−0.71	.478
Resting DBP (mmHg)	76.7 (8.7)	77.6 (8.4)	0.53	.596
Resting HR (bpm)	66.5 (10.8)	65.3 (10.3)	−0.56	.575
Peak SBP (mmHg)*	190 (4)	180 (3)	3.53	.063
Peak DBP (mmHg)*	88 (2)	86 (1)	0.78	.380
Peak HR (mmHg)*	130 (4)	126 (2)	0.72	.399
Peak RPE	17.0 (2.1)	16.8 (1.9)	0.52	.606
Peak R	1.02 (0.08)	1.03 (0.12)	0.43	.670
% Had chest pain (*n*)	50 (17)	55 (45)	0.23	.632
Peak pain rating (Borg scale)	2.3 (2.7)	2.7 (2.8)	0.58	.560
% Max test (*n*: >85% predicted HR)	12 (4)	22 (18)	1.62	.203

*Corrected M (SE), adjusting for resting levels.

ETT: exercise tolerance test, VO_2_: oxygen consumption, SBP: systolic blood pressure, DBP: diastolic blood pressure, HR: heart rate, RPE: Borg rating of perceived exertion.

**Table 3:** Endothelial function variables and ischemia predicting peak oxygen consumption (VO_2_) during exercise testing, adjusting for vessel size, medication status during the FMD test, age, and BMI.

Variable	Model 1		Model 2		Model 3		Model 4	
	*b*	*P*	*b*	*P*	*b*	*P*	*b*	*P*
Ischemia	−0.02	.860	−0.12	.433	−0.04	.970	−0.31	.189
FMD	0.04	.006	−0.01	.866	—	—	—	—
FMD × ischemia	—	—	0.03	.382	—	—	—	—
GTNMD	—	—	—	—	0.02	.019	−0.01	.546
GTNMD × ischemia	—	—	—	—	—	—	0.02	.151

FMD: flow-mediated dilatation, GTNMD: GTN-mediated dilatation

Note: value labeled *b* is the unstandardized regression coefficient from mixed model. Ischemia was scored as ischemia = 1 and no-ischemia = 2. Dash indicates that the variable was not included in the model. Model 1 refers to main effect of FMD and ischemia on peak VO_2_, Model 2 refers to their interaction effects on peak VO_2_, Model 3 represents the main effects of GTNMD and ischemia on peak VO_2_, and Model 4 represents their interaction effects on peak VO_2_.
